# Body Movement Synchrony Predicts Degrees of Information Exchange in a Natural Conversation

**DOI:** 10.3389/fpsyg.2020.00817

**Published:** 2020-04-29

**Authors:** Ayaka Tsuchiya, Hiroki Ora, Qiao Hao, Yumi Ono, Hikari Sato, Kohei Kameda, Yoshihiro Miyake

**Affiliations:** ^1^School of Computing, Tokyo Institute of Technology, Yokohama, Japan; ^2^Department of Computer Science, Tokyo Institute of Technology, Yokohama, Japan

**Keywords:** body movement synchrony, natural conversation, optical motion capture system, exchanging information, motion energy analysis

## Abstract

Human interaction has two principle functions: building and maintaining relationships with others and exchanging information. The function of building and maintaining relationships with others relates to interpersonal coordination; this behavior pattern is expected to predict the outcome of social relationships, such as between therapists and patients. It is unclear, however, whether the exchange of information is associated with interpersonal coordination. In the present study, we tested a hypothesis of whether body movement synchrony occurs in a natural conversation and whether this synchrony has a positive correlation with the degree of information exchange. Fifty participants were engaged in a conversation task; each had different roles in the conversation. We measured their body movements during this conversation using an optical motion capture system. Similar to methods that can be found in previous research, we calculated body movements and quantified their synchrony applying the methods previously reported that automatically quantified their body movements. Moreover, we determined the participants’ degree of information exchange concerning the conversation using a questionnaire. We observed that the body movement synchrony of pairs who talked with each other was significantly higher than that of pairs who did not talk with each other, and that this synchrony was positively associated with the degree of information exchange. These results suggest that body movement synchrony predicted information exchange.

## Introduction

Human beings live in groups and interact with others in daily life through linguistic and non-verbal behaviors. Social interactions involve various communicative forms, such as cooperation, competition, and more (Hari and Kujala, 2009, pp. 453–479). Building cooperative relationships with others is important for belonging to groups and for survival. The need to belong is related to the motivation for human behavior such as cognitive process, emotional patterns, health, and well-being (Baumeister and Leary, 1995, pp. 497–529). Furthermore, group survival depends on maintaining relationships harmoniously within groups; individuals who successfully cooperate with others can obtain their own advantage for survival (De Waal, 1989; Caporael and Brewer, 1991, pp. 187–195; Lakin et al., 2003, pp. 145–162). Thus, human interaction has played an important role in the survival of human beings as social animals.

The reported relationship with interpersonal coordination in human groups is that it leads to the better cohesion and maintenance of social and affective space (Cornejo et al., 2017; Mu et al., 2018). Interpersonal coordination is “the degree to which the behaviors in an interaction are non-random, patterned, or synchronized in both timing and form” (Bernieri, 1988, pp. 120–138). This phenomenon includes various interpersonal behavior patterns, such as behavior matching, mimicry, and synchrony (Cornejo et al., 2017); it is proposed that these interpersonal patterns were supported by mirror neuron system (Gallese et al., 2004, pp. 396–403; Rizzolatti and Craighero, 2004, pp. 169–192; Marsh et al., 2009, pp. 320–339). These patterns are related with liking (Chartrand and Bargh, 1999, pp. 893–910), affiliation (Lakin and Chartrand, 2003, pp. 334–339; Hove and Risen, 2009, pp. 949–960), and rapport (Lakin and Chartrand, 2003, pp. 334–339; Lakens and Stel, 2011, pp. 1–14). For example, humans unintentionally mimic the behaviors of one’s interaction partners (“the chameleon effect”). This mimicry arguably facilitates the smoothness of interactions and increases liking between the interaction partners (Chartrand and Bargh, 1999, pp. 893–910). Hove and Risen demonstrated that synchrony leads to affiliation through a task of synchronous tapping to a metronome (Hove and Risen, 2009, pp. 949–960). Furthermore, the synchronizing of the rhythm of a waving hand increased attributed rapport and perceived entitativity (Lakens and Stel, 2011, pp. 1–14). These studies were in experimental settings but the results suggest that the forms or temporal synchrony of body movement enhances social connection. Furthermore, recent psychological studies, which analyzed interpersonal synchronization in a natural conversation, extended past findings. One of these methods is that quantified body movement is calculated automatically from the amount of change in luminance between recorded video frames or from frequency [e.g., Motion Energy Analysis (Kupper et al., 2010, pp. 90–100; Ramseyer and Tschacher, 2011, pp. 284–295), Motion Energy Detection (Grammer et al., 1999, pp. 284–295), Frame Differencing Methods (Paxton and Dale, 2013a, b, pp. 2092–2102, 329–343), and unnamed method (Nagaoka and Komori, 2008, pp. 1634–1640)]. These methods analyzed interpersonal synchrony in a naturalistic setting. For instance, in psychotherapy, non-verbal synchrony positively correlated with therapeutic relationship quality and self-efficacy, as self-reported by patients (Ramseyer and Tschacher, 2011, pp. 284–295) and in client–counselor relationships (Nagaoka and Komori, 2008, pp. 1634–1640). Thus, synchronization in a naturalistic setting was considered to predict the outcome of people’s interactions and social relationships.

Similarly to non-verbal behavior, verbal behavior is an important aspect of human interaction. Information is exchanged via explicit messages utilizing language and shared representation, to bring understanding with each other. Hasson et al. (2012, pp. 114–121), argued that humans can convey information across brains by verbal behavior, unrelated to the current external environment. They also suggested that brain-to-brain coupling can constrain and shape actions in a social network. Thus, exchanging information also plays a key role in human interaction. Recent cognitive neuroscience studies found a relationship between neural activities and the exchanging information, as well as one between neural activities and body movements. These found that the brain activities of speaker and listener, recorded during verbal communication, are synchronized (Stephens et al., 2010, pp. 14425–14430; Silbert et al., 2014, pp. E4687–E4696). Furthermore, they found that “the greater the extent of neural coupling between a speaker and listener the better the understanding” (Stephens et al., 2010, pp. 14425–14430). Inter-brain synchronization is also suggested as being linked to speech rhythm (Kawasaki et al., 2013). In a study about the relationship between brain activity and body movement, the synchrony of both fingertip movement and brain activity between two participants increased after the experiment, in which one participant followed another participant’s finger movement with their own finger (Yun et al., 2012, p. 959). As these results show, relationships have been reported among neural activity, the degree of information exchange, and speech rhythm, as well as between neural activity and body movement synchrony; body movement synchrony was thus expected to predict the degree of information exchange. However, previous studies, which suggested that synchrony predicts interaction outcome, did not discuss how much information has been exchanged. Thus, the relationship between body movement synchrony and exchanging information is still unclear.

In the present study, we hypothesized that the body movement synchrony between interacting partners may occur during a natural conversation, and that this synchrony may have a positive correlation with the degree of information exchange. To test our hypothesis, we measured body movements and quantified the synchrony of those body movements, then calculated the correlation between the synchrony and degree of information exchange. Body movements were measured by an optical motion capture system. The present study analyzed head movement because the head movements that are related to therapy outcomes are similar to those after exchanging information (Ramseyer and Tschacher, 2014, p. 979). Hence, we analyzed head movement synchrony. The degree of information exchange was assessed with multiple-choice questions with four possible answers.

## Materials and Methods

### Subjects

Participants in this study were 30 females and 20 males healthy right-handed native Japanese speakers (mean age: 27.1 years). All participants met each other for the first time in the experiment. Participants were assigned into 10 groups with 5 in each group. One group, consisted of five participants, was excluded from the analysis because of a problem with measuring body movements. Thus, we reported the results of 45 participants (28 females). This experiment was approved by the ethical committee of Tokyo Institute of Technology, and all participants provided written informed consent.

### Task and Procedure

**FIGURE 1:**
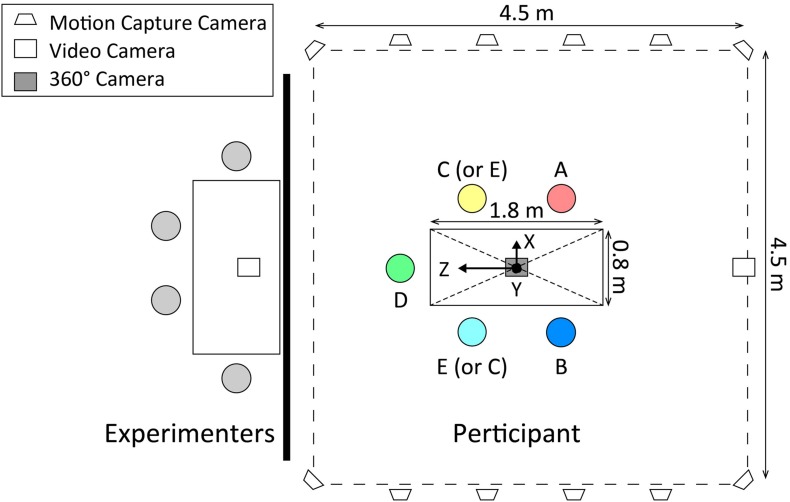
The experimental set-up. Five participants sat around the table. We divided the room by a dark screen so that the participants could not see four experimenters that were preparing the questions. Motion capture cameras were located around the table.

**FIGURE 2:**
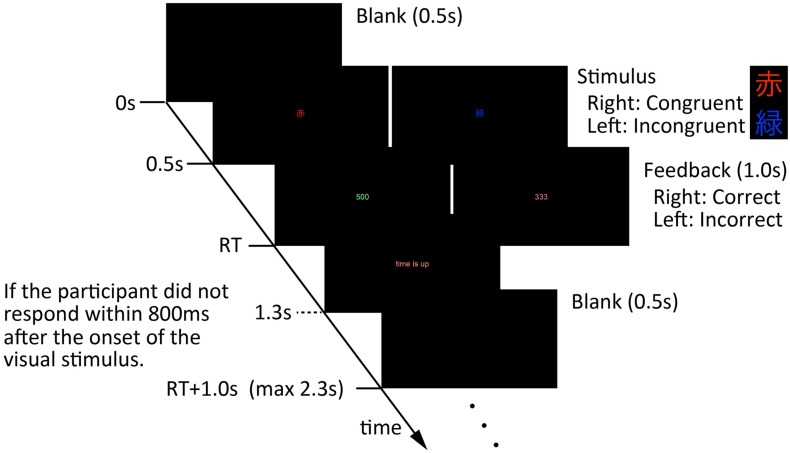
Description of the cognitive task for participant D. Participant D was required to rapidly press a key corresponding to a visual stimulus displayed on the screen of a notebook PC as soon as possible. The visual stimulus used in this task had six patterns; three patterns had congruent letter color and meaning (“red–red,” “green–green,” and “blue–blue”) and three patterns had incongruent letter color and meaning (“red–green,” “green–blue,” and “blue–red”). All stimuli were in Japanese.

The experiments consisted of two sessions. The first session was a behavior session, in which participants engaged in a 10-min conversation; in the second session, the participants answered a questionnaire about the conversation to rate their degree of information exchange. This questionnaire consisted of four-choice questions and we defined the degree of information exchange as a correct answer rate of the questions. Between the sessions, all participants rested for 30 min, which included answering some questions. In the behavior session, the five participants sat around a table (Figure 1). During the conversation, we measured the participants’ body movements. Humans perceive the behavior of others, then unconsciously mimic their behavior (Chartrand and Bargh, 1999, pp. 893–910). Thus, to investigate whether body movement synchrony occurred between interacting partners in an environment in which people who did not talk with others who were there, we assigned five participants (one group) to four roles. We assigned two participants (participants A and B) the role of talking with an interacting partner (participant A or B); the other three participants (participants C, D, and E) were assigned to other roles. All participants played only one role and participated in only one experiment. Participants A and B talked with each other for 10 min. They were instructed to start the conversation by talking about an experience of when they were a freshman in high school, then developed talking based on their experiences. Additionally, they were instructed to assume that their names were “A” and “B” in the conversation. Participant C was instructed to observe the conversation, but was prohibited from talking with participants A and B. Participant D was instructed to observe the conversation and to perform a cognitive task (Figure 2) that used a Stroop effect (Stroop, 1935, pp. 643–662), while she/he observed the conversation. The cognitive task demanded that participant D rapidly press a key corresponding to the color of a letter displayed on the screen of a notebook PC as soon as possible. The stimulus letters were six patterns; three patterns were congruent between letter color and meaning (“red–red,” “green–green,” and “blue–blue”) and three patterns were incongruent between letter color and meaning (“red–green,” “green–blue,” and “blue–red”). All stimuli were in Japanese. Participant D was required to press the left arrow key if it was red, the up one arrow if it was green, and the right arrow if it was blue. This cognitive task had 0.8-s time limits per stimulus and a 10-s interval time per 8 stimuli. When participant D pressed a key, their reaction time was displayed. The reaction time was displayed in green if participant D pressed the correct key, and displayed in red if participant D pressed an incorrect key. If more than 0.8 s passed without participant D pressing any key, a “time is up” message was displayed and this session was recorded as a non-response. After the reaction time was displayed, a blank screen was displayed for 0.5 s, before the next stimulus was displayed (the next session). Each eight sessions, this blank time change to 10 s.

Participant D practiced this task before the behavior session. The role of participant D was prepared for other research; therefore, this paper will not discuss this role in detail. Participant E was a negative control for the degree of information exchange. To shut out visual and audio stimuli, he/she wore headphones, earplugs, and an eye mask. During the conversation, we played white noise in the headphones. To confirm whether participant E was able to derive audio stimulus from the conversation, we instructed participants A and B to talk about “today’s breakfast” and participant E reported whether he/she heard any sentences. We assigned these roles to participants randomly. The roles were counterbalanced by sex. During each conversation, a sheet with the letters of the alphabet corresponding to the participants’ roles was on the table and participants could see this sheet. After we confirmed the roles for the participants, we explained to them that they needed to answer questions about the story of this conversation after the session, and we instructed them to remember this conversation to answer the test as correctly as possible.

After this session, the participants took a 30-min break, which included answering a questionnaire evaluating their impressions of the others. During the break time, we instructed the participants not to use smartphones or talk with the others. In the rating session, the participants answered questions about their conversation during the behavior session. There was no time limit for answering the questions, but we instructed them to answer these questions in order as quickly as possible. We instructed participant E not to answer randomly, but to answer by guessing with common sense as much as possible.

### Measurement

During the behavior session, we measured body movements using an optical motion capture system (VENUS3DR, Nobby Tech. Ltd., Tokyo). Twelve cameras (Flex13, OptiTrack) were located in an area 4.5 m × 4.5 m so that they included the area of the participants sitting around the table. These cameras were placed for measuring the upper limbs of the five participants, who sat around a table (width: 1.79 m, depth: 0.79 m, height: 0.70 m). Participants A and B, who talked with each other, sat facing each other. Participant C, who observed conversation, and participant E, who was a negative control, sat next to participants A and B. Participant D, who performed the cognitive task, sat between participants C and E. In front of participant D was a notebook PC and a keyboard, which was used for the cognitive task. Both participants and experimenters were in the same room for the behavior session. The four experimenters stayed outside of the measurement area of the motion capture system, where the participants could not see because it was divided by a dark screen (Figure 1).

**FIGURE 3:**
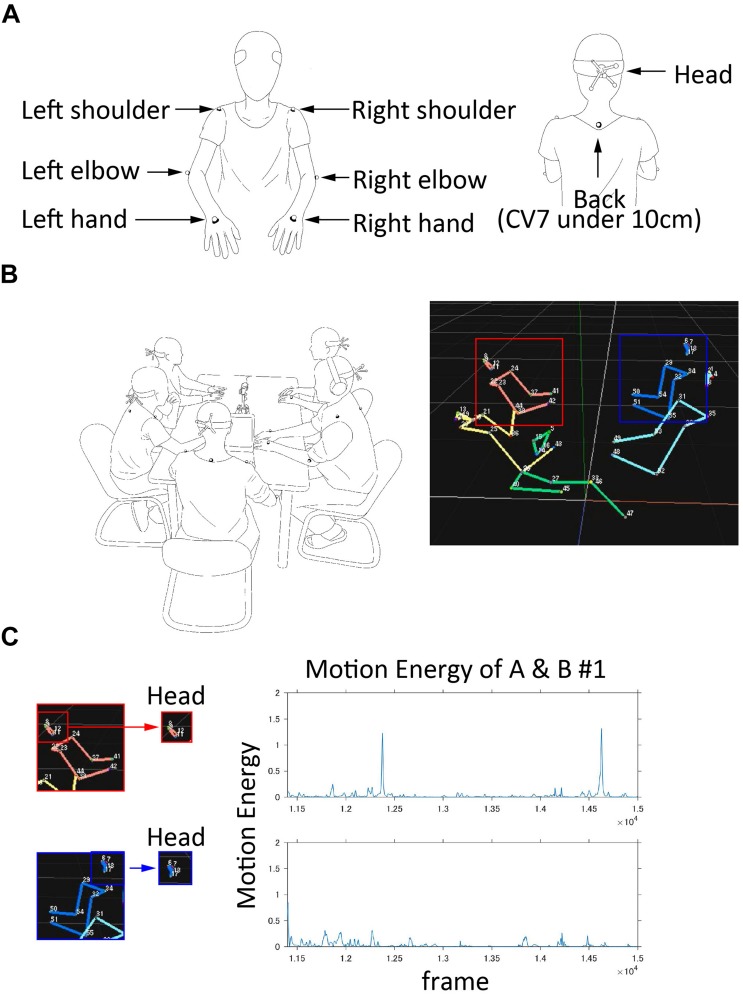
**(A)** The attached placement of infrared reflection markers. **(B)** Left: Original video frame recorded in present experiment. Right: A frame reconstructed by the 3D coordinates of the infrared reflection markers. Participant A is shown as a red square and participant B is shown as a blue square. **(C)** Time series data of head movement. This was calculated as the sum of squared speed of the four markers attached to each participant’s head in each frame. The vertical axis is the sum of squared speed, and the horizontal axis is the frame. Using this data, we analyzed body movement synchrony.

We recorded body movements using software called Motive (v1.9.0, OptiTrack) at 120 frames per second. This optical motion capture system measures the three-dimensional (3D) coordination of infrared reflection markers, which reflect the infrared light emitted by the cameras. The Motive software calibrates all infrared camera positions and recognizes and records a marker’s position by capturing reflected areas. Conventional stereo video analysis may reduce the information with regard to the depth direction, but this system can obtain movements in three-dimensional coordination, including depth. Infrared reflection markers were attached to 11 points per person (4 points of the head, 2 points of the left and right acromion, 2 points of the lateral epicondyle of the humerus, 2 points of the left and right hands, and 1 point of 10 cm below the 7th cervical vertebra); we therefore recorded the 3D coordinates of 55 points per experiment. The coordinate system of the motion capture system was a left-handed system, with an origin 5 mm above the surface of the table’s center. Participants wore a headset that attached four infrared reflection markers on their heads. A marker base was placed at the rear of this headset, with four markers attached. The marker placement pattern of each participant’s headset was different. We did not use a marker suit, because such equipment may interfere with the natural movements and/or conversations of the participants. A same-sex experimenter attached the other markers on the participants’ upper bodies either over the participants’ clothes or on their skin (Figure 3A) using medical tape. In the case of attaching the markers over participants’ clothes, we fixed the clothing to prevent it from shifting these markers. After the participants were seated around the table, but before the conversation, we confirmed that all 55 markers were recognized by the motion capture system. In this study, we used only the head markers for the analysis; the other markers will be used for another research. In addition, we recorded the session using a 360° camera (Kodak PIXPRO SP360 4K, Jk Imaging, Ltd., Los Angeles) that was placed on the center of the table, and two video cameras (SONY HANDYCAM HDR-CX270 and FDR-AX40, Sony Corporation, Tokyo) that were placed from behind participant D and to the side to provide a profile view of participants A and B (Figure 1). The videos recorded by the 360° camera will be used in another research, while the other videos recorded by the two side video cameras were used to prepare the questions for rating the degree of information exchange. Figure 3B shows an original video frame and a reconstructed frame from the captured 3D coordinates of the infrared reflection markers.

### Quantification of Body Movement Synchrony

We analyzed body movements using sums of squared velocity, as referred to in Motion Energy Analysis (Ramseyer and Tschacher, 2011, pp. 284–295). The VENUS3DR optical motion capture system recorded the 3D trajectory of the infrared reflection markers attached to each body part. Sometimes, each measured trajectory recorded was divided into two or more trajectories. To adjust these divided trajectories into one actual trajectory, we manually labeled the markers using the VENUS3DR software, and visually inspected the results. After this preprocessing, we calculated the velocity time series data from each of the four head markers’ trajectories, using the VENUS3DR software. In this process, the speed of any missing data that failed to follow a marker was replaced with 0 speed. We analyzed the time series data, which were calculated as the sum of squared speed time series data from the speed time series data of the four head markers (Figure 3C).

**FIGURE 4:**
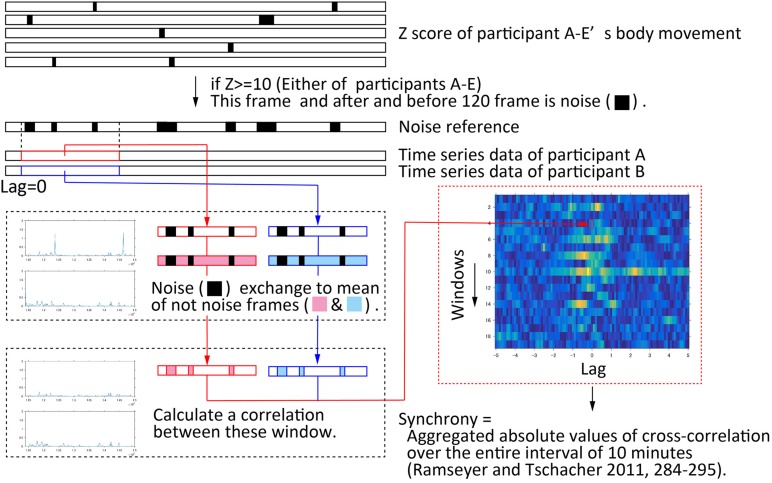
Procedure of quantifying body movement synchrony. First, time series data of the sum of squared speed of the four head markers was calculated for each participant. Second, large accidental movements were excluded from this time series data. To exclude these movements, if the Z score of any of participant A–E’s movement was greater than 10, it was determined that this frame, and 120 frames before and after (= 1 s), was noise. To ensure that the removal of noise did not affect any correlation, the noise in the window was padded with the average value of the non-noise section in the window. The color map represents the values of cross-correlation. The window is on the vertical axis. The lag is on the horizontal axis, and steps up from 0 s to ± 5 s by 0.1 s. The average of all correlation coefficients obtained over the entire 10-min interval was the body movement synchrony in this conversation.

To quantify the body movement synchrony, the time series of the sum of squared speed were cross-correlated (Boker et al., 2002, pp. 338–355; Ramseyer and Tschacher, 2011, pp. 284–295) in window segments of 30 s duration, and step-wise shifted 30 s (Tschacher et al., 2014, p. 1323). Cross-correlations were calculated stepped up from 0 s to ± 5 s by 0.1 s (50 steps) (Tschacher et al., 2014, p. 1323). We excluded body movements that were large accidental movements. We calculated a Z score of each participant using time series data, and defined noise as a movement where the Z score was more than 10. To avoid an influence of the other participants’ noise, we calculated a disjunction of noises of all participants in the same behavior session. In addition, we excluded 120 frames before and after noise, because of body movements being consecutive. To avoid an influence for calculating cross-correlation, we replaced any data of noise from a mean of non-noise movements in this window. We used a global synchrony, which was the aggregated absolute values of cross-correlation over the entire 10-min interval (Ramseyer and Tschacher, 2011, pp. 284–295). Figure 4 shows the procedure of this quantitative analysis.

### Quantification of Information Exchange

The degree of information exchange was rated by the score of four-choice questions about the 10-min conversation held by participants A and B in the behavior session. The questionnaire was prepared by four experimenters during the behavior session (10 min) and resting time (30 min). To develop the questionnaire, four experimenters referred to the 10-min conversation in real time as well as to the video recording of this conversation. Four experimenters were in charge of preparing the questions (one experimenter for each quarter of the conversation, lasting 2 min and 30 s), and all experimenters were instructed to prepare more than 10 questions insomuch as it was possible. The number of experimenters and questions were decided through preliminary experiments. All questions were based on sentences that participants A and B had uttered in the conversation. Typical examples of these questions were “Did person A say…?” and “Where did person B go to high school?” The questions were ordered by the time in which they were spoken, and were provided for the participants in the relevant group.

## Results

### Measurement

This resulted in a total of nine groups (*N* = 9: three groups with a mixed-sex interacting pair, four groups with two females in the pair, and two groups with two males in the pair). We analyzed body movement synchrony by measuring 1 to 72,000 frames; on average, 64,379.8 frames were actually used for each analysis (on average, 7620.2 frames were excluded because of noise). The questionnaire used in the second session was created for each experiment by four experimenters, and the mean value of the number of the questions created was 41.3.

### Behavior

**FIGURE 5:**
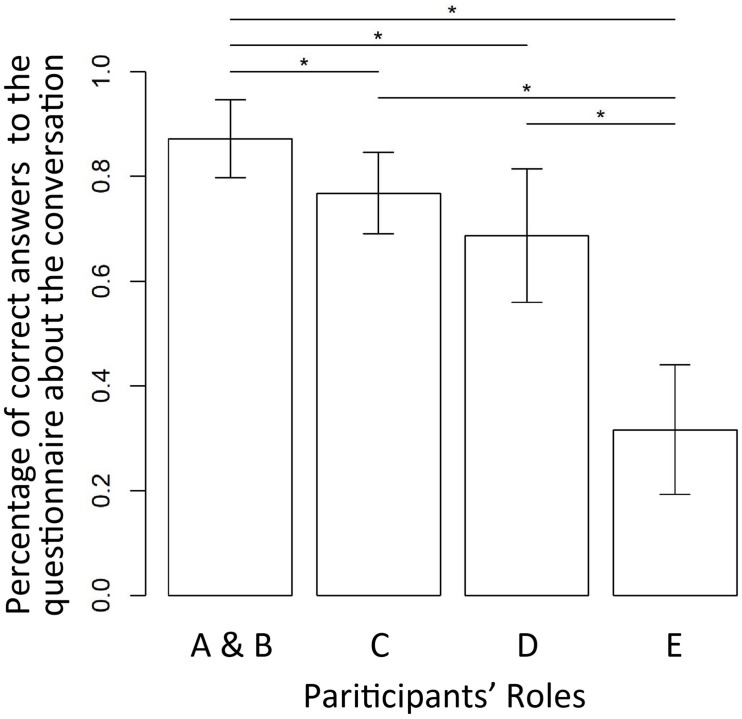
Percentage of information exchange showing significant difference between each role, except roles C and D. The information exchange of roles A and B was significantly higher than that of the other roles, and the information exchange of roles E scored significantly lower than the roles who listened to the conversation (roles C and D). ^∗^Indicate significant difference at *p* < 0.05.

The behavior results showed that the degree of information exchange of roles A and B, who interacted with each other, scored 87.2% (*SD* = 7.48); that of role C, who observed only, scored 76.8% (*SD* = 7.78); that of role D, who observed and performed the Stroop task, scored 68.7% (*SD* = 12.7); and that of role E, who was the negative control for exchanging information, scored 31.7% (*SD* = 12.4) (Figure 5).

The degree of information exchange of roles A and B (who interacted each other) was significantly higher in comparison to each of the other roles (Steel-Dwass test, AB–C: *t* = 2.68, *p* = 0.037, AB–D: *t* = 3.29, *p* = 0.0055, AB–E: *t* = 4.17, *p* = 0.00018). In comparison with role E, who was the negative control, each of the other roles was significantly higher (AB–E: *t* = 4.17, *p* = 0.00018, C–E: *t* = 3.58, *p* = 0.0020, D–E: *t* = 3.49, *p* = 0.0027).

### Body Movement Synchrony

**FIGURE 6:**
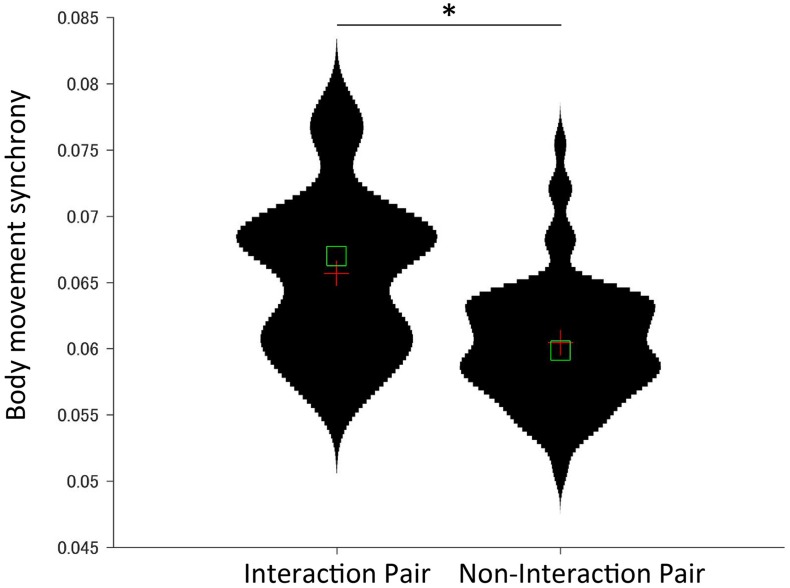
Body movement synchrony of interacting and non-interacting pairs were significantly different. Interacting pairs (9 pairs) were participants A and B from the same experiments. Non-interacting pairs (72 pairs) were combined participants A and B who participated in different sessions (i.e., they did not talk with each other). ^∗^Indicate significant difference at *p* < 0.05.

To confirm whether body movement synchrony occurred between the two people who interacted with each other, we tested the comparison of 9 AB pairs who actually talked within the pair and 72 AB pairs who participated in different behavior sessions, using Wilcoxon rank-sum test (Figure 6). The result showed that these two groups were significantly different (*p* = 0.014, Cliff’s delta = −0.51).

### Body Movement Synchrony Predicts Degrees of Information Exchange in Natural Conversation

**FIGURE 7:**
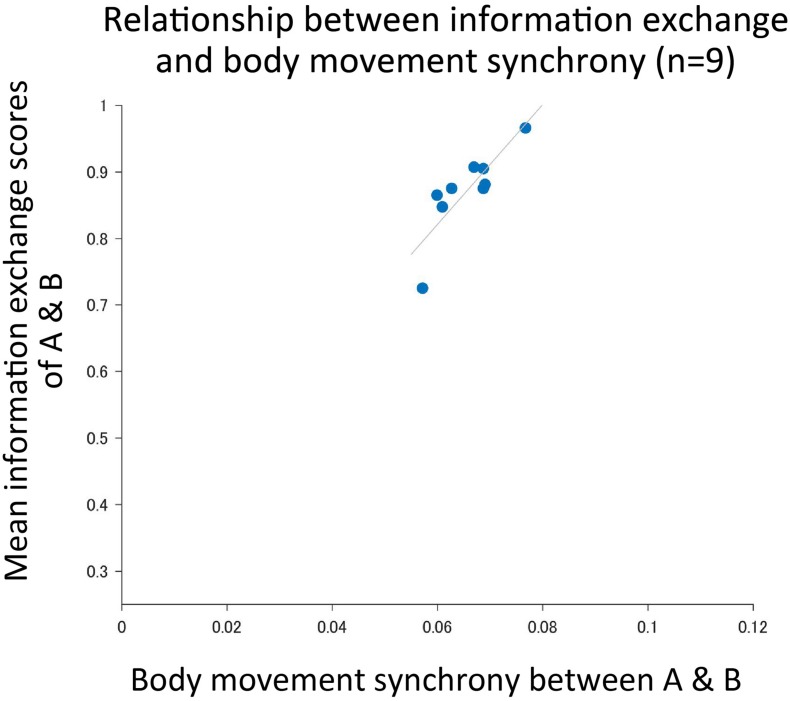
The body movement synchrony that occurred in interacting pairs was greater with the degree of information exchange. The horizontal axis is the amount of body movement synchrony, and the vertical axis is the average of the degree of information exchange of participants A and B. Each plotted point is the result of one experiment (*n* = 9).

To test whether the body movement synchrony that occurred between two people who interacted with each other was correlated to the degree of information exchange, we calculated Pearson’s correlation coefficient, and found a positive correlation (*r* = 0.84, *p* = 0.0046) (Figure 7). The retrospective power analysis (α = 0.05, two-tails) regarding the correlation of Pearson’s r resulted in 1-β = 0.89.

## Discussion

We hypothesized that body movement synchrony may occur between interacting partners during a natural conversation, and that this synchrony may have a positive correlation with the degree of information exchange. To test this hypothesis, we measured body movement and quantified synchrony, and calculated the correlation between this synchrony and the degree of information exchange. The results revealed that body movement synchrony occurred in interacting partners during a natural conversation, and that this synchrony was positively correlated with the degree of information exchange.

The present task in behavior session seems to be appropriate to enact natural conversation. We quantified the degree of information exchange during natural conversations with different contents in each experiment. In this study, two participants engage in a non-rehearsed natural conversation based on their experiences. We instructed participants only to begin talking about an experience they had had when they were freshmen in high school, and to develop the conversation based on those experiences as much as possible. Throughout all of the experiments, participants were able to continue their conversation for 10 min without silence. Therefore, the instructions given by the experimenter were appropriate to develop a conversation between participants who were meeting each other for the first time in the experiment.

The degree of information exchange of roles A and B, who talked with each other, was significantly higher than the degree of information exchange for each role of C, D, and E. In particular, the degree of information exchange for roles A and B (87.2%, *SD* = 7.48) was significantly higher than the degree of information exchange for role E, who shut out visual and audio stimuli as a negative control for exchanging information (31.7%, *SD* = 12.4). In comparison with the other role’s degree of information exchange (those who observed the conversation), role E’s degree of information exchange was significantly lower. This result suggested that the questionnaire used in the study rated the degree of information exchange appropriately. Additionally, the degree of information exchange of roles A and B was significantly higher than that of role C. This result was considered to occur from the fact that role A and B had their own information but role C did not have any of their information. This fact indicated that the questionnaire, which rated the degree of information exchange, accurately reflected the contents of the story told by roles A and B in the behavior session. These results indicated that roles A and B exchanged information about their experience.

When comparing the results of body movement synchrony, the synchrony of the two people who interacted with each other was significantly higher than the synchrony of pairs who did not talk within the pair. This result was consistent with the results of previous studies, which investigated body movement synchrony in a natural conversation (Ramseyer and Tschacher, 2011, pp. 284–295; Latif et al., 2014, p. e113316). This indicated that body movement synchrony occurred in interacting partners during a natural conversation. Additionally, the pairs who did not talk within the pair did not share a space, so their condition was similar to participant E, who was shut out of visual and audio stimuli as a negative control for the degree of information exchange. Both the degree of information exchange and body movement synchrony were significantly higher than those of the negative control. Therefore, this result suggested that body movement synchrony and degree of information exchange both increased by participants interacting with another.

The relationship between body movement synchrony and the degree of information exchange had a positive correlation, and this result supports our hypothesis. The present result showed that the body movement synchrony of the interacting pair, who shared their personal experiences, was greater than the synchrony of the non-interacting pair, who did not share personal information with each other. This is consistent with the result of a previous study, in which the synchrony between friends who know personal information about each other is higher than the synchrony between strangers (Latif et al., 2014, p. e113316). This result therefore suggested that body movement synchrony predicted the degree of information exchange. Moreover, body movement synchrony was expected be an objective indicator for improving a conversation in real time.

The present result showed that body movement synchrony predicts not only social relationships but also the degree of information exchange. It suggested that head movement synchrony may be related to exchanging information. Ramseyer et al., reported that head- and body- movement synchrony is correlated with different assessments in counseling sessions (Ramseyer and Tschacher, 2014, p. 979). Thus, the synchrony of different body parts is considered to reflect a different characterization of interaction outcomes, such as exchanging information and social relationships, and that humans may evaluate their overall interaction outcomes by integrating these body movement synchronies. In the future, additional use of psychological tests may help to provide further insight for body movement synchrony.

In this study, however, the causal direction of information exchange and body movement synchrony remains unclear. If there was a causality relation, we would be able to discuss different aspects of body movement synchrony. If body movement synchrony would lead to the degree of information exchange; for instance, our hypothesis would support that body movement synchrony may affect social cognitive function (Miles et al., 2010, pp. 457–460). For example, social connection, which is enhanced by body movement synchrony, is expected to decrease the advantage of self-memory (Symons and Johnson, 1997, pp. 371–394); this effect may then cause a greater exchanging of information. On the other hand, if the degree of information exchange would lead to body movement synchrony, we may obtain evidence of the mechanism for how body movement synchrony occurs. Especially, body movement synchrony and brain activity increased after a cooperative task (Yun et al., 2012, p. 959); therefore, the degree of inter-brain synchrony may be reflected in the amount of body movement synchrony. In the future, examining these hypotheses will facilitate discussion about how such a mechanism of body movement synchrony occurs, and its effect on social cognition.

The present study found that body movement synchrony occurred in interacting partners during a natural conversation, and that this synchrony predicted the degree of information exchange.

### Limitations

The present study has some limitations. Firstly, we examined only nine experiments. It is necessary to test a larger sample size for more reliable results. Secondly, the measurement was concerned to affect body movements. To measure body movements, we used an optical motion capture system. The three-dimensional coordination of head positions were recognized by infrared reflection markers attached to a headset worn by the participants. In addition, markers attached to the participants’ upper bodies, either over their clothes or on their skin using surgical tape. However, these markers may have affected their body movements.

## Data Availability Statement

The datasets generated for this study are available on qualified request to the corresponding author.

## Ethics Statement

The studies involving human participants were reviewed and approved by the Ethical Committee of Tokyo Institute of Technology. The patients/participants provided their written informed consent to participate in this study.

## Author Contributions

AT and HO analyzed the data and wrote the manuscript. HO and YM designed the research. AT, QH, YO, HS, and KK performed the research.

## Conflict of Interest

The authors declare that the research was conducted in the absence of any commercial or financial relationships that could be construed as a potential conflict of interest.
